# Network partitioning algorithms as cooperative games

**DOI:** 10.1186/s40649-018-0059-5

**Published:** 2018-10-28

**Authors:** Konstantin E. Avrachenkov, Aleksei Y. Kondratev, Vladimir V. Mazalov, Dmytro G. Rubanov

**Affiliations:** 1 Inria Sophia Antipolis, 2004 Route des Lucioles, 06902 Valbonne, France; 2 Higher School of Economics, 16 Soyuza Pechatnikov St., St. Petersburg, 190121 Russia; 3 Institute of Applied Mathematical Research, Karelian Research Center, Russian Academy of Sciences, 11 Pushkinskaya St., Petrozavodsk, 185910 Russia; 4 Saint-Petersburg State University, 7/9 Universitetskaya Nab., St. Petersburg, 199034 Russia

**Keywords:** Network partitioning, Community detection, Cooperative game, Myerson value, Hedonic game, Gibbs sampling

## Abstract

The paper is devoted to game-theoretic methods for community detection in networks. The traditional methods for detecting community structure are based on selecting dense subgraphs inside the network. Here we propose to use the methods of cooperative game theory that highlight not only the link density but also the mechanisms of cluster formation. Specifically, we suggest two approaches from cooperative game theory: the first approach is based on the Myerson value, whereas the second approach is based on hedonic games. Both approaches allow to detect clusters with various resolutions. However, the tuning of the resolution parameter in the hedonic games approach is particularly intuitive. Furthermore, the modularity-based approach and its generalizations as well as ratio cut and normalized cut methods can be viewed as particular cases of the hedonic games. Finally, for approaches based on potential hedonic games we suggest a very efficient computational scheme using Gibbs sampling.

## Introduction

Community detection in networks is a very important topic which has numerous applications in social network analysis, computer science, telecommunications, and bioinformatics and has attracted the effort of many researchers. In the present work, we consider the framework of crisp community detection or network partitioning, where one would like to partition a network into disjoint sets of nodes. The consideration of overlapping, hierarchical, and local clustering we leave for future research. Even the literature on crisp community detection is huge. We refer to several extensive survey papers [1–6]. Let us just mention main classes of methods for network partitioning. The first very large class is based on spectral elements of the network matrices such as adjacency matrix and Laplacian (see e.g., the surveys [1, 5] and references therein). The second class of methods is based on the use of random walks (see e.g., [7–12] for the most representative works in this research direction). The third class of approaches to network partitioning is based on the optimization of some objective function [13–19], with modularity function [15, 16] as a notable example in this category. Finally, the fourth class, directly related to the present work, is based on the notions from game theory. We recommend to an interested reader a recent survey [4] on the application of game-theoretic techniques to community detection. Most bibliography described in [4] is in fact dedicated to non-cooperative game theory approaches. It appears that the application of the cooperative or coalition games to community detection problem is under-developed and thus with this article we advance this research area.

There are definitely many relations among the above-mentioned classes. In particular, the conditions for minima of the objective functions can often be interpreted in terms of the eigen elements of the network matrices. The eigen elements of the network matrices also characterize the stationary or quasi-stationary state of a random walk on a network. In the present work, we show more connections between the approach based on cooperative games and other approaches.

In essence, all the above-mentioned approaches, with exception of the game theory approach, try to detect dense subgraphs inside the network and do not address the question: what are the natural forces and dynamics behind the formation of network clusters. As noticed in [20], most of traditional clustering methods pursue a top-down approach, whereas typically communities are formed by local interactions in self-organizing fashion, often driven by egocentric decisions. Thus, it is very natural to apply game theory, and in particular, coalition game theory for community detection problem. Also, in most of the above-mentioned methods, the number of communities is a prerequisite parameter. The game theory approach typically does not require a priori knowledge of the number of communities. One more very important benefit in using the methods from game theory is that such methods are naturally distributed and can easily be implemented in clouds and decentralized multi-agent systems.

In the present work, we explore two cooperative game theory approaches to explain possible mechanisms behind cluster formation. Our first approach is based on the Myerson value in cooperative game theory, which particularly emphasizes the value allocation in the context of games with interactions between players constrained by a network. The advantage of the Myerson value is in taking into account the impact of all coalitions. We extend the method developed in [21, 22] to calculate efficiently the Myerson value in a network. A number of network centrality measures based on game-theoretic concepts have been developed, see [22–28] and references therein. It might be interesting to combine node ranking and clustering based on the same approach such as the Myerson value to analyze the network structure. Unfortunately, the computation of the Myerson value is a very difficult problem even for a moderately large number of players. Therefore, we propose the second approach which has efficient computational implementation and can easily be distributed.

The second approach is based on hedonic games [29], which are games explaining well the mechanism behind the formation of coalitions. Both our approaches allow to detect clusters with varying resolutions and thus avoiding the problem of resolution limit [30, 31]. The hedonic game approach is especially well suited to adjust the level of resolution as the limiting cases are given by the grand coalition and sequential maximum clique decomposition, two very natural extreme cases of network partitioning. Furthermore, the modularity-based approaches as well as ratio cut [32] and normalized cut [10, 33] based methods can be cast in the setting of hedonic games. We find that this gives one more, very interesting, interpretation of the modularity-based methods. The advantage of casting the ratio cut and normalized cut in the framework of hedonic games is that we do not need to prespecify the number of clusters as was needed in the original formulations of these methods.

Some hierarchical network partitioning methods based on tree hierarchy, such as [15], cannot produce a clustering on one resolution level with the number of clusters different from the predefined tree shape. Furthermore, the majority of clustering methods require the number of clusters as an input parameter. In contrast, in our approaches we specify the value of the resolution parameter(s) and the method gives a natural number of clusters corresponding to the given resolution parameter(s).

Let us point out major differences between our approaches and approaches suggested in the other works on cooperative game theory for network clustering. In [34], a cooperative game theory approach based on Shapley value has been proposed. However, with the proposed characteristic function, the players tend to form the grand coalition. In the subsequent work [35], a new characteristic function has been proposed, which combines both link-based as well as attribute-based information. The Shapley value associated with that characteristic function is very cumbersome to compute in comparison to the Myerson value for the characteristic function proposed in the first part of our paper. Of course, we admit that the computation of any type of Shapley value is computationally demanding and this is why we propose the second approach which has an efficient, naturally distributed, computational implementation.

The authors of [20] have also proposed to use hedonic games for community detection. They consider only the modularity metric as value function. They have suggested an additional voting mechanism to overcome the resolution problem. Their algorithm is a version of greedy optimization. Our approach is much more general: not only we show that the modularity optimization is a particular case of our approach but we also demonstrate that such known methods as ratio cut and normalized cut are also particular cases of our approach. We also propose a couple of new functions that overcome the resolution problem without a need of additional voting mechanism. Our Gibbs sampling-based algorithm can be used with both fixed and decreasing temperature and hence can be used for local as well as global maxima search. Setting the temperature to a very low value corresponds to the greedy approach.

The authors of [36] in the first part of their paper propose to use the concept of strong Nash equilibrium in addition to the concept of hedonic games. They also define a community as a $(\lambda ,\gamma )$-relaxation of the clique. There are several serious problems with their propositions. First of all, the strong Nash equilibrium might not exist (they acknowledge this fact themselves in their work), and such equilibrium is very hard to compute even if it exists. Furthermore, they give two definitions of a maximal $(\lambda ,\gamma )$-relaxation of the clique which are contradictory and therefore their algorithm can cycle.

We also note that our approaches based on cooperative games easily work with multi-graphs, where several edges (links) are possible between two nodes. A multi-edge has several natural interpretations in the context of social networks. A multi-edge can represent a number of telephone calls; a number of exchanged messages; a number of common friends; or a number of co-occurrences in some social event.

Let us now summarize the main contributions of the paper (we place$^*$ in the items, which are new additions to the work in comparison with the conference version [37]):

- First the cooperative game theory approach based on the Myerson value is proposed for network partitioning.
- Then the hedonic coalition formation framework is proposed for network partitioning which has more efficient computational implementation than the approach based on the Myerson value.
- New interpretation in terms of hedonic games is given to modularity, ratio cut, and normalized cut network partitioning methods.$^*$
- Two new network partitioning methods based on potential hedonic games are proposed. (One method is a new addition with respect to the conference paper [37].)$^*$ These two methods are especially well suited to find partitions with different levels of resolution; the methods use only one or two parameters. We provide recommendations how to set these parameters.
- For methods constructed on potential hedonic games, we suggest to use a very efficient computational algorithm based on Gibbs sampling.$^*$
- Several numerical evaluations using real$^*$ as well as synthetic networks are carried out. These numerical evaluations in particular demonstrate the efficacy of the clustering methods based on potential hedonic games with resolution regularization.

The paper is structured as follows: in the following section, we provide necessary definitions from graph theory, network partitioning, and network games. Then, in “Myerson cooperative game approach” section, we present our first approach based on the Myerson value. The second approach based on the hedonic games is presented in “Hedonic coalition game approach” section. In both “Myerson cooperative game approach and Hedonic coalition game approach” sections, we provide small illustrative examples to explain the essence of the methods. In “Numerical validation” section, we evaluate our methods on a number of real as well as synthetic network examples. Finally, “Conclusion and future research” section provides conclusions and directions for future research.

## Preliminaries of graph theory, network partitioning, and network stability

Let $g=(N,E)$ denote an undirected multi-graph consisting of the set of nodes *N* and the set of edges *E*. We denote an edge (link) between node *i* and node *j* as *ij*. The interpretation is that if $ij\in E$, then the nodes $i\in N$ and $j\in N$ have a direct connection in network *g*, while $ij\notin E$, then nodes *i* and *j* are not directly connected. Since we generally consider a multi-graph, there could be several edges between a pair of nodes. Multiple edges can be interpreted for instance as a number of telephone calls or as a number of message exchanges in the context of social networks.

We view the nodes of the network as players in a cooperative game. Let $N(g)=\{i:\exists j \text{ such } \text{ that } ij\in E(g)\}$. For a graph *g*, a sequence of different nodes $\{i_1,i_2,\dots ,i_k\},\ k\ge 2$, is a path connecting $i_1$ and $i_k$ if for all $h=1,\dots ,k-1$, $i_hi_{h+1}\in g$. The length *l* of a path is the number of edges in that path, i.e., $l=k-1$. A path with no repeated nodes is called a *simple path*. Graph *g* on the set *N* is connected graph if for any two nodes *i* and *j* there exists a path in *g* connecting *i* and *j*.

We refer to a subset of nodes $S \subset N$ as a coalition. The coalition *S* is connected if any two nodes in *S* are connected by a path which consists of nodes from *S*. The graph $g'$ is a (connected) component of *g*, if for all $i\in N(g')$ and $j\in N(g')$, there exists a path in $g'$ connecting *i* and *j*, and for any $i\in N(g')$ and $j\in N(g)$, $ij\in g$ implies that $ij\in g'$. Let *N*|*g* be the set of all (connected) components in *g* and let *g*|*S* be the subgraph with the nodes in *S*.

Let $g-ij$ denote the graph obtained by deleting edge *ij* from the graph *g* and $g+ij$ denote the graph obtained by adding edge *ij* to the graph *g*.

The result of community detection is a partition of the network (*N*, *E*) into subsets (coalitions) $\{S_1,\ldots ,S_K\}$ such that $S_k \cap S_l=\emptyset , \forall k,l$ and $S_1\cup ...\cup S_K=N$. This partition is *internally stable* or *Nash stable* if for any player from coalition $S_k$ it is not profitable to join another (possibly empty) coalition $S_l$. We also say that the partition is *externally stable* if for any player $i\in S_l$ for whom it is beneficial to join a coalition $S_k,$ there exists a player $j\in S_k$ for whom it is not profitable to include there player *i*. The payoff definition and distribution will be discussed in the following two sections.

## Myerson cooperative game approach

In general, a cooperative game of *n* players is a pair $<N,v>$ where $N=\{1,2,\ldots ,n\}$ is the set of players and *v*: $2^N\rightarrow R$ is a map prescribing for a coalition $S\in 2^N$ some value *v*(*S*) such that $\textit{v}(\emptyset ) = 0$. This function *v*(*S*) is the total utility that members of *S* can jointly attain. Such a function is called the characteristic function of cooperative game. An interested reader can find more details on cooperative games in e.g., [38–40].

Additionally, as in [41], we assume that the cooperation is restricted by a network. The payoff to an individual player is called an imputation. The imputation specifies how the value associated with the network is distributed to the individual players. The imputation in our cooperative game will be based on the Myerson value [21, 22, 41] which was designed to take into account the effect of the network.

The Myerson value [41] is the allocation rule

$$\begin{aligned} Y(v,g)=(Y_1(v,g), \ldots , Y_n(v,g)), \end{aligned}$$

where $Y_i(v,g)$ is the payoff allocated to player *i* from graph *g* under the characteristic function *v*. The Myerson value is uniquely determined by the following two axioms [41]:

### **Axiom 1**

If *S* is a connected component of *g*, then the members of the coalition *S* ought to allocate to themselves the total value *v*(*S*) available to them, i.e., $\forall S\in N|g$,

1 $$\begin{aligned} \sum _{i\in S} Y_i(v, g)=v\left( S\right) . \end{aligned}$$

### **Axiom 2**

$\forall g,\ \forall ij\in g$ both players *i* and *j* obtain equal payoffs after adding or deleting a link *ij*,

2 $$\begin{aligned} Y_i\left( v,g\right) -Y_i\left( v,g-ij\right) =Y_j\left( v,g\right) -Y_j\left( v,g-ij\right) . \end{aligned}$$

Characteristic function (payoff of coalition *S*) can be defined in different ways. Here we use a general idea from [21, 22, 42, 43], which is based on discounting paths. However, unlike [21, 22, 42, 43], we do not consider shortest paths but rather *simple paths*.

Let us elaborate a bit more on the construction of the characteristic function. Each edge (or direct connection) gives to coalition *S* the value *r*, where $0\le r \le 1$. Moreover, players obtain a value from indirect connections. Namely, each *simple* path of length 2 belonging to coalition *S* gives to this coalition the value $r^2$, a simple path of length 3 gives to the coalition the value $r^3$, etc. Set

$$\begin{aligned} v(i)=0,\ \forall i\in N. \end{aligned}$$

Thus, for any coalition *S*, we can define the characteristic function as follows:

3 $$\begin{aligned} v(S) = a_1(g, S) r + a_2(g, S) r^2 + \cdots = \sum _{k = 1}^{\infty } a_k(g, S) r^k, \end{aligned}$$

where $a_k(g, S)$ is the number of simple paths of length *k* in this coalition. Note that we write as infinity the limit of summation only for convenience. Clearly, the length of a simple path is bounded by $n-1$. The following theorem provides a convenient way to calculate the Myerson value corresponding to the characteristic function (3).

### **Theorem 1**

*Let the characteristic function of a coalition* $S \in 2^N$ *be defined by Eq.* (3). *Then the Myerson value of a node* *i* *is given by*

4 $$\begin{aligned} Y_i(v, g) = \frac{a_1^{(i)}(g, S)}{2} r + \frac{a_2^{(i)}(g, S)}{3} r^2 + \cdots = \sum _{k = 1}^{\infty } \frac{a_k^{(i)}(g, S)}{k+1} r^k, \end{aligned}$$

*where* $a_k^{(i)}$ *is the number of simple paths of length* *k* *containing node* *i*.

### *Proof*

We shall prove the theorem by checking directly the Myerson value axioms, i.e., Axioms 1 and 2.

First, we note the following:

$$\begin{aligned} (k+1)a_k(g, S) = \sum _{i \in S} a_k^{(i)}(g, S). \end{aligned}$$

Since every simple path contains $k+1$ different nodes, every simple path of the length *k* is counted $k+1$ times in the sum $\sum _{i \in S} a_k^{(i)}(g, S)$.

Thus, Axiom 1 is satisfied:

$$\begin{aligned} \sum _{i \in S} Y_i(v, g) = \sum _{i \in S} \sum _{k = 1}^{\infty } \frac{a_k^{(i)}(g, S)}{k+1} r^k = \sum _{i \in S} a_k(g, S) r^k = v(S). \end{aligned}$$

For $i j \in g$, let $a_k^{(i j)}(g, S)$ denote the number of paths of length *k* traversing the edge *ij*. Then

$$\begin{aligned} a_k^{(i)}(g, S) - a_k^{(i)}(g - i j, S) = a_k^{(i j)}(g, S) = a_k^{(j)}(g, S) - a_k^{(j)}(g - i j, S) \end{aligned}.$$

Thus, Axiom 2 is satisfied as well. $\square$

We can propose the following algorithm for network partitioning based on the Myerson value: Start with a partition of the network $N=\{1,\ldots ,n\}$, where each node forms her own coalition. Consider a coalition $S_l$ and a player $i\in S_l$. In the cooperative game with partial cooperation presented by the graph $g|S_l,$ we find the Myerson value for player *i*, $Y_i(g|S_l)$. This is the reward of player *i* in coalition $S_l$. Suppose that player *i* decides to join the coalition $S_k$. In the new cooperative game with partial cooperation presented by the graph $g|S_k\cup i,$ we find the Myerson value $Y_i(g|S_k\cup i)$. So, if for the player $i\in S_l:$ $Y_i(g|S_l)\ge Y_i(g|S_k\cup i)$ then player *i* has no incentive to join to new coalition $S_k$, otherwise the player changes the coalition.

The partition $N=\{S_1,\ldots ,S_K\}$ is the Nash stable or internally stable if for any player there is no incentive to move from her coalition. Notice that our definition of the characteristic function implies that for any coalition it is always beneficial to accept a new player (of course, for the player herself it might not be profitable to join that coalition). Thus, it is important that in the above algorithm, we consider the internal and not external stability. If one makes moves according to the external stability, then the result will always be the grand coalition.

We would like to note that the above approach also works in the case of multi-graphs, where several edges (links) are possible between two nodes. In such a case, if two paths contain different links between the same pair of nodes, we consider these paths as different.

### *Example 1*

**Fig. 1:**
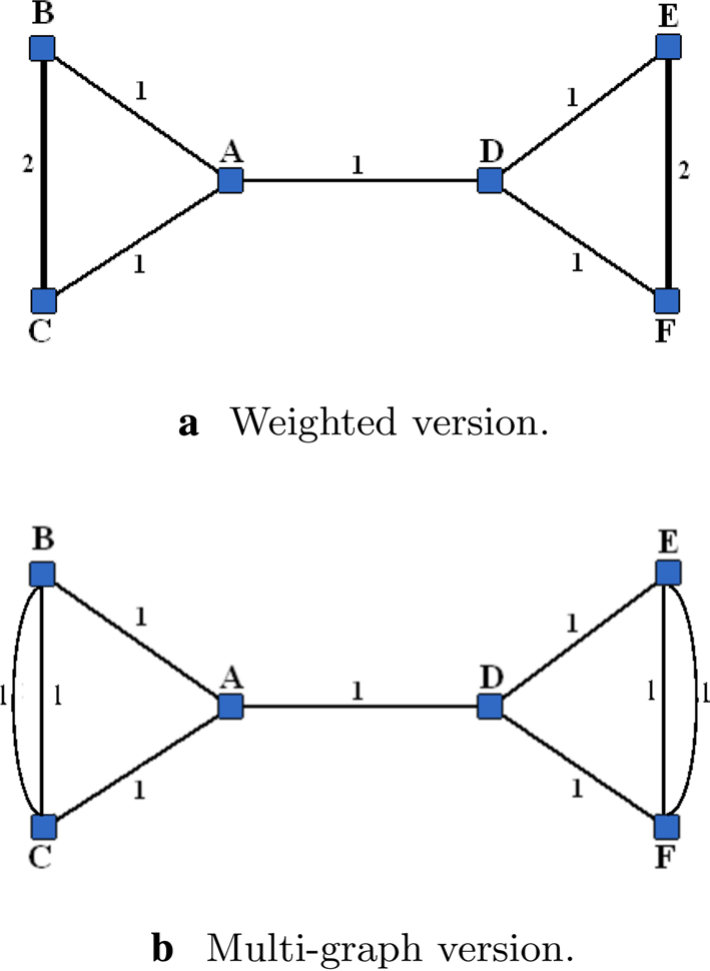
Network of six nodes

Consider a weighted network of six nodes, $N=\{A,B,C,D,E,F\}$, presented in Fig. 1a. First, we transform this weighted graph to the multi-graph as shown in Fig. 1b.

A natural way of partition of this network is $\{S_1=\{A,B,C\}, S_2=\{D,E,F\}\}$. Let us determine under which condition this structure will present the internally stable partition.

Suppose that the characteristic function is defined by (3). To calculate the imputations, we use the formula (4) from Theorem 1. In the coalition $S_1$, node *A* participates in 2 simple paths of length 1 $\{\{A,B\}, \{A,C\}\}$ and in 5 simple paths of length 2 $\{\{A,B,C\},\{A,B,C\},\{A,C,B\},\{A,C,B\},\{B,A,C\}\}$ (note that since the network example is a multi-graph we count twice the paths {A,B,C} and {A,C,B}). Thus, we have

$$\begin{aligned} Y_A(g|S_1) = \frac{2}{2} r + \frac{5}{3} r^2. \end{aligned}$$

In the coalition $S_2 \cup A$, node *A* participates in 1 simple path of length 1 $\{A,D\}$, in 2 simple paths of length 2 $\{\{A,D,E\}, \{A,D,F\}\}$ and in 4 simple paths of length 3 $\{\{A,D,E,F\}, \{A,D,E,F\}, \{A,D,F,E\}, \{A,D,F,E\}\}$ (again these paths are counted twice for the multi-graph). Thus,

$$\begin{aligned} Y_A(g|S_2 \cup A) = \frac{1}{2} r + \frac{2}{3} r^2 + \frac{4}{4} r^3. \end{aligned}$$

We see that for player *A* it is not profitable to move from $S_1$ to $S_2 \cup A$, if

$$\begin{aligned} \frac{1}{2} r + r^2 - r^3 > 0, \end{aligned}$$

which is valid for all *r* in the interval (0, 1]. Therefore, in this partition node, *A* has no incentive to change the coalition under any choice of *r*.

**Fig. 2:**
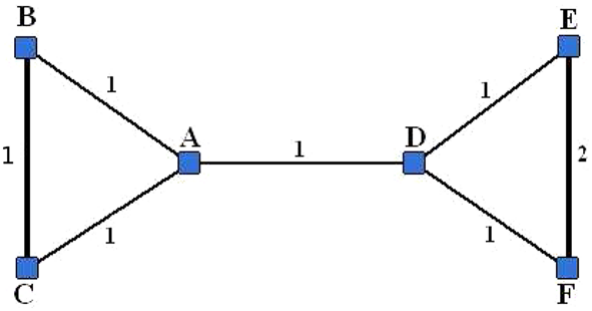
Network of six nodes with changed weight

Now consider a slightly modified example, where we change the weight 2 on the edge $\{B,C\}$ to weight 1 (see Fig. 2). This change results in the following imputations:

$$\begin{aligned} Y_A(g|S_1) = \frac{2}{2} r + \frac{3}{3} r^2, \end{aligned}$$

and

$$\begin{aligned} Y_A(g|S_2 \cup A) = \frac{1}{2} r + \frac{2}{3} r^2 + \frac{4}{4} r^3. \end{aligned}$$

We see that now $Y_A(g|S_2 \cup A) > Y_A(g|S_1)$, if $r > (1+\sqrt{19})/6 \approx 0.89$. Thus, if *r* is sufficiently large, the partition $\{S_1,S_2\}$ becomes internally unstable and the grand coalition becomes the only stable configuration.

In the above example the parameter *r* can be used to tune the resolution of network partitioning. Resolution scale tuning will be even more natural in the next approach. We shall see that the next approach is also much more computationally efficient than the Myerson value-based approach.

## Hedonic coalition game approach

There is another game-theoretic approach for partitioning society into coalitions based on the ground-breaking work [29] on hedonic games.

Assume that the set of players $N=\{1,\ldots , n\}$ is divided into *K* coalitions by the partition $\Pi =\{S_1,\ldots ,S_K\}$. Let $S_\Pi (i)$ denote the coalition $S_k\in \Pi$ such that $i\in S_k$. A hedonic game is defined in terms of player preferences for various coalitions. A player *i* preferences are represented by a complete, reflexive, and transitive binary relation $\succeq _i$ over the set $\{S\subset N: i\in S\}$. Denote by $\succ _i$ the strict part of this relation.

Let us now apply the framework of hedonic games [29] to network partitioning problem, particularly, specifying the preferences. First, in the next subsection, we consider the case of additively separable preferences and then in “The case of non-additively separable preferences” section, we consider the case of non-additively separable preferences.

### The case of additively separable preferences

The preferences are additively separable [29] if there exists a value function $v_i:N\rightarrow \mathbb {R}$ such that $v_i(i)=0$ and

$$\begin{aligned} S_1\succeq _i S_2 \Leftrightarrow \sum \limits _{j\in S_1}{v_i(j)}\ge \sum \limits _{j\in S_2}{v_i(j)}. \end{aligned}$$

The preferences $\{v_i, i\in N\}$ are symmetric, if $v_i(j)=v_j(i)=v_{ij}=v_{ji}$ for all $i,j\in N$. The symmetry property defines a very important class of hedonic games.

As in the previous section, the network partition $\Pi$ is *Nash stable*, if $S_\Pi (i)\succeq _i S_k\cup \{i\}$ for all $i\in N, S_k\in \Pi \cup \{\emptyset \}$. In the Nash-stable partition, there is no player who wants to leave her coalition.

A potential of a coalition partition $\Pi =\{S_1,\ldots ,S_K\}$ (see [29]) is

5 $$\begin{aligned} P(\Pi )=\sum _{k=1}^{K} P(S_k)=\sum \limits _{k=1}^K{\sum \limits _{i,j\in S_k}{v_{ij}}}. \end{aligned}$$

One natural method for detecting a stable community structure can be based on the following better response type dynamics:

Start with any partition of the network $N=\{S_1,\ldots ,S_K\}$. Choose any player *i* and any coalition $S_k$ different from $S_\Pi (i)$. If $S_k\cup \{i\} \succ _i S_\Pi (i)$, assign node *i* to the coalition $S_k$; otherwise, keep the partition unchanged and choose another pair of node-coalition, etc.

Since the game has the potential (5), the above algorithm is guaranteed to converge in a finite number of steps.

#### **Proposition 1**

*If players’ preferences are additively separable and symmetric* ($v_{ii}=0, v_{ij}=v_{ji}$ *for all* $i,j\in N$), *then the coalition partition* $\Pi$ *giving a local maximum of the potential* $P(\Pi )$ *is the Nash-stable partition.*

One natural way to define a symmetric value function *v* with a parameter $\alpha \in [0,1]$ is as follows:

6 $$\begin{aligned} v_{ij}= \left\{ \begin{array}{ll} 1-\alpha , &{} (i,j)\in E, \\ -\alpha , &{} (i,j)\notin E, \\ 0, &{} i=j. \end{array} \right. \end{aligned}$$

For any subgraph *g*|*S*, denote the number of nodes in *S* as *n*(*S*), and the number of edges in *S* as *m*(*S*). Then, for the value function (6), the potential (5) takes the form

7 $$\begin{aligned} P^\alpha (\Pi )=\sum \limits _{k=1}^K{\left( m(S_k) - \alpha \frac{n(S_k)(n(S_k)-1)}{2} \right) }. \end{aligned}$$

We can characterize the limiting cases $\alpha \rightarrow 0$ and $\alpha \rightarrow 1$. Towards this goal, let us introduce a special decomposition of the network into cliques. At first, let us find a maximum clique $S_1$ in the network *G* (a maximum clique of a graph, is a clique, such that there is no clique with more vertices). Remove all vertices of $S_1$ from *G* and consider the new network $G'$. Let us find a maximum clique $S_2$ in the network $G'$ and continue this procedure until we derive the partition $\{S_1,...,S_K\}$ of the network *G* into cliques. Call this partition the sequential decomposition of the network into maximum cliques.

#### **Proposition 2**

*If* $\alpha =0$, *the grand coalition partition* $N=\{1,\ldots ,n\}$ *gives the maximum of the potential* (7). *Whereas if* $\alpha \rightarrow 1$, *the network sequential decomposition into maximum cliques corresponds to a maximum of the potential* (7).

#### *Proof*

It is immediate to check that for $\alpha =0$ the grand coalition partition *N* gives the maximum of the potential (7), and $P^\alpha (N)=m(N)$.

For values of $\alpha$ closed to 1, the partition into maximum cliques $\Pi =\{S_1,\ldots ,S_K\}$ gives the maximum of (7). Indeed, assume that a player *i* from the clique $S_{\Pi }(i)$ of the size $m_1$ moves to a clique $S_j$ of the size $m_2<m_1$. The player $i\in S_{\Pi }(i)$ and $S_j$ are connected by at most $m_2$ links. The impact on $P^\alpha (\Pi )$ of this movement is not higher than

$$\begin{aligned} m_2(1-\alpha )-(m_1-1)(1-\alpha )\le 0. \end{aligned}$$

Now, suppose that player *i* from the clique $S_{\Pi }(i)$ moves to a clique $S_j$ of the size $m_2\ge m_1$. Notice that clique $S_j$ was constructed in the procedure of sequential decomposition before the clique $S_{\Pi }(i)$. The player $i\in S_{\Pi }(i)$ is connected with the clique $S_j$ by at most $m_2-1$ links. Otherwise, the clique $S_j$ can be increased by adding node *i* and this contradicts the fact that $S_j$ was a maximum clique at the procedure of decomposition. If *i* has an incentive to move from $S_{\Pi }(i)$ to the clique $S_j$, then for new partition the sum (7) would not be higher than for partition $\Pi$ by

$$\begin{aligned} m_2-1-m_2\alpha -(m_1-1)(1-\alpha )=m_2-m_1-\alpha (m_2-m_1+1). \end{aligned}$$

For $\alpha$ close to 1, this impact is negative, so there is no incentive to join the coalition $S_j$. $\square$

The grand coalition and the sequential maximum clique decomposition are two extreme partitions into communities. By varying the parameter $\alpha$ we can easily tune the resolution of the community detection algorithm.

#### *Example 2*

**Fig. 3:**
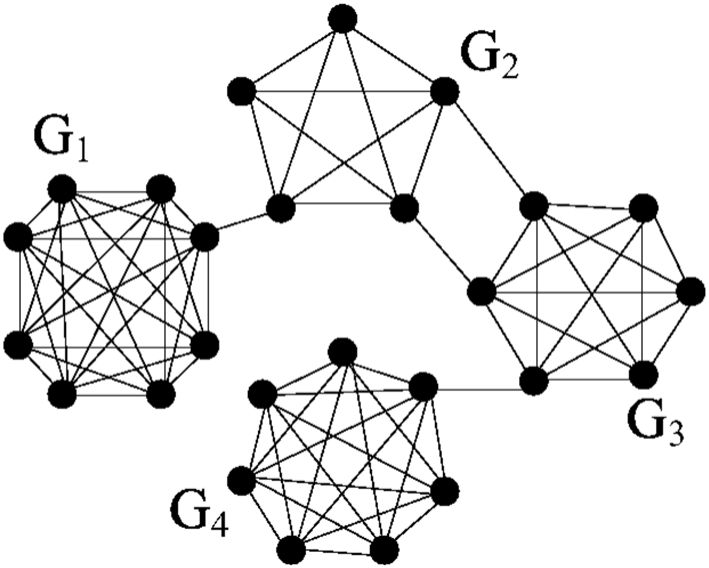
Graph with four fully connected subgraphs

Consider graph $G=(N,E)$, which consists of $n=26$ nodes and $m=78$ edges (see Fig. 3). This graph includes 4 fully connected subgraphs: $G_1$ with 8 vertices $N_1$ connected by 28 links, $G_2$ with 5 vertices $N_2$ connected by 10 links, $G_3$ with 6 vertices $N_3$ connected by 15 links, and $G_4$ with 7 vertices $N_4$ connected by 21 links. Subgraph $G_1$ is connected with $G_2$ by 1 edge, $G_2$ with $G_3$ by 2 edges, and $G_3$ with $G_4$ by 1 edge.

Firstly, calculate the potentials (7) for large-scale decompositions of *G* for any parameter $\alpha \in [0,1]$. It is easy to check, that $P(N)=78-325\alpha$, $P(\{N_1, N_2\cup N_3\cup N_4\})=77-181\alpha$, $P(\{N_1, N_2\cup N_3, N_4\})=76-104\alpha$, $P(\{ N_1, N_2, N_3, N_4\} )=74-74\alpha$.

Other coalition partitions give smaller potentials: $P(\{ N_1 \cup N_2, N_3\cup N_4\})=76-156\alpha <76-104\alpha$, $P(\{ N_1 \cup N_2 \cup N_3, N_4\})=77-192\alpha <77-181\alpha$, $P(\{ N_1, N_2, N_3 \cup N_4 \})=75-116\alpha <76-104\alpha$, $P(\{ N_1 \cup N_2, N_3, N_4 \})=75-114\alpha <76-104\alpha$.

We solve a sequence of linear inequalities in order to find maximum of the potential for all $\alpha \in [0,1]$. The result is presented in the table.

**Table:** Nash-stable coalition partitions in Example 2

$\alpha$	Coalition partition	Potential
[0, 1/144]	$N_1 \cup N_2 \cup N_3 \cup N_4$	$78-325\alpha$
[1/144, 1/77]	$N_1, N_2 \cup N_3 \cup N_4$	$77-181\alpha$
[1/77, 1/15]	$N_1, N_2 \cup N_3, N_4$	$76-104\alpha$
[1/15, 1]	$N_1, N_2, N_3, N_4$	$74-74\alpha$

*Example 1 (ctnd)* Note that for the unweighted version of the network example presented in Fig. 1, there are only two stable partitions: $\Pi =N$ for small values of $\alpha \le 1/9$ and $\Pi =\{\{A,B,C\},\{D,E,F\}\}$ for $\alpha >1/9$.

Another natural approach to define a symmetric value function is, roughly speaking, to compare the network under investigation with the configuration random graph model. The configuration random graph model can be viewed as a null model for a network with no community structure. Namely, the following value function can be considered:

8 $$\begin{aligned} v_{ij}=\beta _{ij}\left( A_{ij}-\delta \frac{d_i d_j}{2m}\right) , \end{aligned}$$

where $A_{ij}$ is the number of links between nodes *i* and *j* (multi-graph is allowed), $d_i$ and $d_j$ are the degrees of nodes *i* and *j*, respectively, $m=\frac{1}{2} \sum _{l\in N} d_l$ is the total number of links in the network, and $\beta _{ij}=\beta _{ji}$ and $\delta$ are some parameters.

Note that if $\beta _{ij}=\beta , \forall i,j \in N,$ and $\delta =1$, the potential (7) coincides with the network modularity [15, 16]. If $\beta _{ij}=\beta , \forall i,j \in N,$ and $\delta \ne 1$, we obtain the generalized modularity presented first in [18]. The introduction of the non-homogeneous weights was proposed in [19] with the following particularly interesting choice:

$$\begin{aligned} \beta _{ij} = \frac{2m}{d_id_j}. \end{aligned}$$

The introduction of the resolution parameter $\delta$ allows one to obtain clustering with varying granularity as well as to overcome the resolution limit [30].

Thus, we now have an interpretation based on coalition game of the modularity method. Namely, the coalition partition $\Pi =\{S_1,\ldots ,S_K\}$ which maximizes the modularity

9 $$\begin{aligned} P(\Pi )=\sum \limits _{k=1}^K{\sum \limits _{i,j\in S_k, i\ne j}\left( A_{ij}-\frac{d_i d_j}{2m}\right) } \end{aligned}$$

gives the Nash-stable partition of the network in the hedonic game with the value function defined by (8) with $\delta =1$ and $\beta _{ij}=\beta$.

*Example 1 (ctnd)* For the network example presented in Fig. 1, we calculate $P(N)=3/2, P(\{B,C\}\cup \{A,D\}\cup \{E,F\})= P(\{A,B,C,D\}\cup \{E,F\})=7/2$ and $P(\{A,B,C\}\cup \{D,E,F\})=5$. Thus, according to the value function (8) with $\delta =1$ and $\beta _{ij}=\beta$ (modularity value function), $\Pi =\{\{A,B,C\},\{D,E,F\}\}$ is the unique Nash-stable coalition partition.

### The case of non-additively separable preferences

Now let us consider a few cases of non-additively separable preferences which still have potentials. First, we consider a slight modification of preference structure (6) which makes it non-additively separable. Namely, define the preference relation as follows:

10 $$\begin{aligned} S_1\succeq _i S_2 \Leftrightarrow \sum \limits _{j\in S_1}{v_{ij}} -\gamma 1\{S_1 \ne \emptyset \} \ge \sum \limits _{j\in S_2}{v_{ij}} -\gamma 1\{S_2 \ne \emptyset \}, \end{aligned}$$

where $1\{\cdot \}$ is the indicator function, giving one if the argument is true, $v_{ij}$ is defined as before in (6), and $\gamma$ is a parameter representing the cost of coalition creation and allows us to control further the clustering resolution and granularity. As verified in the following proposition, in this case the game also has a potential.

#### **Proposition 3**

*The hedonic clustering game defined by the preference relation* (10) *has the following potential:*

11 $$\begin{aligned} P^{\alpha ,\gamma }(\Pi )=\sum \limits _{k=1}^K{\left( m(S_k) -\alpha \frac{n(S_k)(n(S_k)-1)}{2} \right) } - \gamma K. \end{aligned}$$

#### *Proof*

Suppose that partition $\Pi =\{S_1,...,S_K\}$ maximizes the function (11), possibly locally. Then, if *i* moves from $S_{\Pi }(i)$ to $S_k$, the value of (11) corresponding to the new partition $\Pi '$ is different from the value corresponding to $\Pi$ by

$$\begin{aligned} \sum \limits _{j\in S_k} v_{ij}-\sum \limits _{j\in S_\Pi (i)} v_{ij}, \end{aligned}$$

note that *K* is not changing. If *i* creates its own cluster, then the value of (11) corresponding to $\Pi '$ is different from that for $\Pi$ by

$$\begin{aligned} -\gamma -\sum \limits _{j\in S_\Pi (i)} v_{ij}, \end{aligned}$$

and in the case $S_{\Pi }(i)=\{i\}$, the difference is

$$\begin{aligned} \sum \limits _{j\in S_k} v_{ij}+\gamma . \end{aligned}$$

If $\Pi$ provides a maximum of (11), all these differences are negative. So, according to relation (10), player *i* indeed has no incentive to move from her coalition $S_{\Pi }(i)$ to another coalition and the function (11) can be interpreted as a potential. $\square$

Let us provide a few recommendations for the choice of $\alpha$ and $\gamma$. Similarly to [18], from the analysis of the mean field model corresponding to a stochastic block model (SBM), one can show that the value of $\alpha$ close to the link density ensures the internal stability of clusters in the mean field model of SBM. Thus, if a network has one main scale, such value of $\alpha$ gives good result. If a network has nested clustering structure, one can vary $\alpha$ to obtain clustering with the needed level of granularity. Again using the mean field model for SBM, one can show that the good value of $\gamma$ corresponds to the product of $\alpha$ and the smallest size of the cluster we would like to obtain.

We mention that interestingly under a specific choice of parameters the globally optimal partition may contain disconnected clusters. However, such a choice of parameters is typically not natural.

**Fig. 4:**
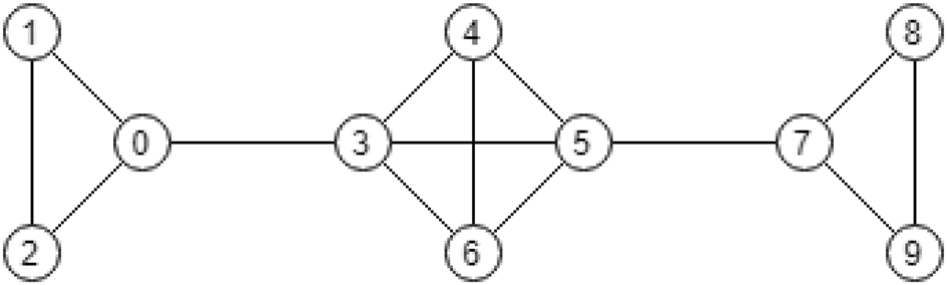
Graph consisting of three cliques

#### *Example 3*

Let us consider a graph that consists of a clique of four nodes and two cliques of three nodes connected to it (see Fig. 4).

One can check that for $\alpha = 0.5$ and $\gamma = 5,$ the partitioning

$$\begin{aligned} \tilde{\Pi } = \{\{0, 1, 2, 7, 8, 9\}, \{3, 4, 5, 6\}\} \end{aligned}$$

gives the maximum value to the potential $P^{\alpha ,\gamma }(\tilde{\Pi }) = -\,8.5$, while

$$\begin{aligned}&P^{\alpha ,\gamma }( \{\{0, 1, 2, 3, 4, 5, 6, 7, 8, 9\}\}) = -\,18.5,\\&P^{\alpha ,\gamma }( \{\{0, 1, 2, 3, 4, 5, 6\}, \{7, 8, 9\}\}) = -\,9,\\&P^{\alpha ,\gamma }( \{\{0, 1, 2\}, \{3, 4, 5, 6\}, \{7, 8, 9\}\}) = -\,9. \end{aligned}$$

An intuitive interpretation for this choice of parameters is that $\alpha$ is chosen significantly large to encourage splitting of clusters from the grand coalition and $\gamma$ is also chosen significantly large to penalize the creation of independent clusters.

Next we would like to note that two well-known network partitioning methods: normalized cut [10, 33] and ratio cut [32] can also be viewed as particular instances of potential hedonic clustering games with non-additively separable preferences. Towards this end, let us introduce a few more definitions. Let $S,T \subset N$ be two, possibly overlapping sets of nodes. Then, we define a cut *W*(*S*, *T*) as

$$\begin{aligned} W(S,T) = \sum _{i \in S, j \in T} 1\{ (i,j) \in E \}. \end{aligned}$$

Note that an edge is counted twice if its both ends lie in the same set. The volume of a set $S \in N$ is defined as the number of edges between its vertices

$$\begin{aligned} {\text vol}(S) = \frac{1}{2} W(S,S). \end{aligned}$$

The normalized cut of a set *S* is defined by

$$\begin{aligned} h^\text{NCUT}(S) = \frac{W(S,\bar{S})}{W(S,S)} = \frac{1}{2} \frac{W(S,\bar{S})}{\text{vol}(S)}, \end{aligned}$$

where $\bar{S}=N\backslash S$. If $\text{vol}(S)=0$, we define $h^\text{NCUT}(S) = +\infty$. One can also define the ratio cut

$$\begin{aligned} h^\text{RCUT}(S) = \frac{W(S,\bar{S})}{|S|}. \end{aligned}$$

Similar to the normalized cut, we assign $h^\text{RCUT}(S) = +\infty$, if $|S|=0$, i.e., $S = \emptyset$. Then, the normalized cut [10, 33] and ratio cut [32] network partitioning methods are based on the following potentials

12 $$\begin{aligned} P^\text{NCUT}(\Pi )= & {} - \sum _{S \in \Pi } h^\text{NCUT}(S), \end{aligned}$$

13 $$\begin{aligned} P^\text{RCUT}(\Pi )= & {} - \sum _{S \in \Pi } h^\text{RCUT}(S), \end{aligned}$$

respectively. Similar to the proof of Proposition 3, one can check that the above potentials correspond to the following preferences: for the normalized cut

14 $$\begin{aligned}&S_1\succeq _i S_2 \Leftrightarrow \nonumber \\&\quad \frac{W(S_1 \bigcup \{i \}, {\bar{S}}_1\backslash \{i\})}{vol(S_1 \bigcup \{i \})} - \frac{W(S_1, {\bar{S}}_1\backslash \{i\})}{vol(S_1)} \le \frac{W(S_2 \bigcup \{i \}, {\bar{S}}_2\backslash \{i\})}{vol(S_2 \bigcup \{i \})} - \frac{W(S_2, {\bar{S}}_2\backslash \{i\})}{vol(S_2)} \end{aligned}$$

and the ratio cut

15 $$\begin{aligned}&S_1\succeq _i S_2 \Leftrightarrow \nonumber \\&\quad \frac{W(S_1 \bigcup \{i \}, {\bar{S}}_1\backslash \{i\})}{|S_1|+1} - \frac{W(S_1, {\bar{S}}_1\backslash \{i\})}{|S_1|} \le \frac{W(S_2 \bigcup \{i \}, {\bar{S}}_2\backslash \{i\})}{|S_2|+1} - \frac{W(S_2, {\bar{S}}_2\backslash \{i\})}{|S_2|}, \end{aligned}$$

respectively. Thus, two more, well-known network partitioning methods can be cast into our general framework. A very important benefit of such interpretation is that in contrast to the original formulations, we now do not require a priori knowledge of the number of clusters.

### Gibbs sampling approach for hedonic games with potential

We note that finding Nash equilibrium in a game with potential is equivalent to finding a maximum of the game’s potential. To find a maximum of the game’s potential, we can follow the approach based on Gibbs sampling. Let us consider the following Gibbsian distribution over all partitions:

16 $$\begin{aligned} \rho (\Pi ) = \frac{\exp \left( \beta P(\Pi )\right) }{\sum _{\forall \tilde{\Pi }} \exp \left( \beta P(\tilde{\Pi })\right) }. \end{aligned}$$

It is easy to see that as $\beta \rightarrow \infty$, the distribution concentrates on the partition corresponding to the maximum of the potential $P(\Pi )$.

Next, denote by $\Sigma$ the set of indices of the network clusters and by $\Pi _{i\rightarrow \sigma }$ the (re)assignment of node *i* to cluster $\sigma \in \Sigma$ and run the Glauber dynamics [18, 44] according to

17 $$\begin{aligned} P_{\Pi \rightarrow \Pi '} = \frac{1}{n} \left\{ \begin{array}{cl} \sum \limits _{i \in N} \frac{\exp \left( \beta P(\Pi ) \right) }{\sum \nolimits _{s\in \Sigma }\exp \left( \beta P(\Pi _{i\rightarrow s}) \right) }, &{} \text {if } \Pi ' = \Pi , \\ \frac{\exp \left( \beta P(\Pi ') \right) }{\sum \nolimits _{s\in \Sigma }\exp \left( \beta P(\Pi _{i\rightarrow s}) \right) }, &{} \text {if } \Pi ' = \Pi _{i \rightarrow \sigma }, \\ 0, &{} \text {otherwise,} \end{array} \right. \end{aligned}$$

that is, we choose randomly a node and reassign this node to a new cluster according to the conditional Gibbsian distribution (clearly, it can happen that the node remains in its current cluster). It is well known that the Glauber dynamics corresponds to the reversible Markov chain with the stationary distribution given by (16), see e.g., [44]. One can also cool down the temperature as in simulated annealing [45] in order to find the partition with the global maximum of the potential. We define one iteration as *n* updates of nodes according to (17). Typically and as will be demonstrated in the next section, if we take a reasonably high inverse temperature $\beta$, the process (17) often finds good-quality partition already after 5–10 iterations. The complexity of one iteration is very light in the case of sparse graphs, i.e., *O*(|*E*|).

A sample generated by the above-described Glauber dynamics appears to have significant variance. To reduce it, the generalized empirical covariance matrix of several samples can be used, similar to [46] where the standard covariance matrix has been used for the case of two clusters. For one sample, the elements of the generalized covariance matrix are defined as follows:

$$\begin{aligned} M^{(1)}_{i,j} = \left\{ \begin{matrix} 1, &{} \text {if } \sigma (i) = \sigma (j) , \\ -1, &{} \text {otherwise.} \end{matrix}\right. \end{aligned}.$$

The empirical generalized covariance matrix of a set of samples is the average of their generalized covariance matrices, i.e.,

$$\begin{aligned} \hat{M} = \frac{1}{T} \sum _{t=1}^{T} M^{(t)}. \end{aligned}$$

An (*i*, *j*)th value of the generalized covariance matrix indicates how often the *i*th and *j*th nodes appear in the same cluster.

Then, given a generalized covariance matrix one can extract the community structure using threshold-based or PCA-based methods.

## Numerical validation

In this section, we validate the proposed approaches on synthetic and real-world networks. As a benchmark, we take a widely used clustering method sklearn.cluster.spectralclustering from [47]. The method is based on the eigen elements of the normalized Laplacian and *K*-means postprocessing and have demonstrated good performance in many previous studies.

If it is available, the ground truth clustering is denoted by $\Pi ^{\text {true}}$ and the clustering obtained by an algorithm as $\Pi ^{\text {test}}$. Each time we specify which algorithm we test against the ground truth. We measure the difference between these two partitions by the following function from [48]:

18 $$\begin{aligned} {\mathcal {E}} (\Pi ^{\text {true}}, \Pi ^{\text {test}}) = 1 - \frac{1}{n} \underset{\pi : \Sigma ^{\text {true}} \rightarrow \Sigma ^{\text {test}}}{\max } \sum _{\sigma \in \Sigma ^{\text {true}}} n_{\sigma , \pi (\sigma )}. \end{aligned}$$

### Synthetic network: stochastic block model

We first evaluate various clustering algorithms based on potential hedonic games on stochastic block model (SBM), a synthetic network with known community structure. An SBM with $|\Sigma |$ clusters is represented by a symmetric square matrix *P* where $p_{\sigma , \sigma }$ is a density of edges inside the cluster $\sigma$ and $p_{\sigma , \sigma '} = p_{\sigma ', \sigma }$ is a density between clusters $\sigma$ and $\sigma '$. Specifically, we use SBM with two communities of 50 and 150 nodes, intra-cluster density $p_{11} = p_{22} = 0.1$ and inter-cluster density $p_{12} = p_{21} = 0.02$. We start from a random coloring and run the process for 100 iterations.

**Fig. 5:**
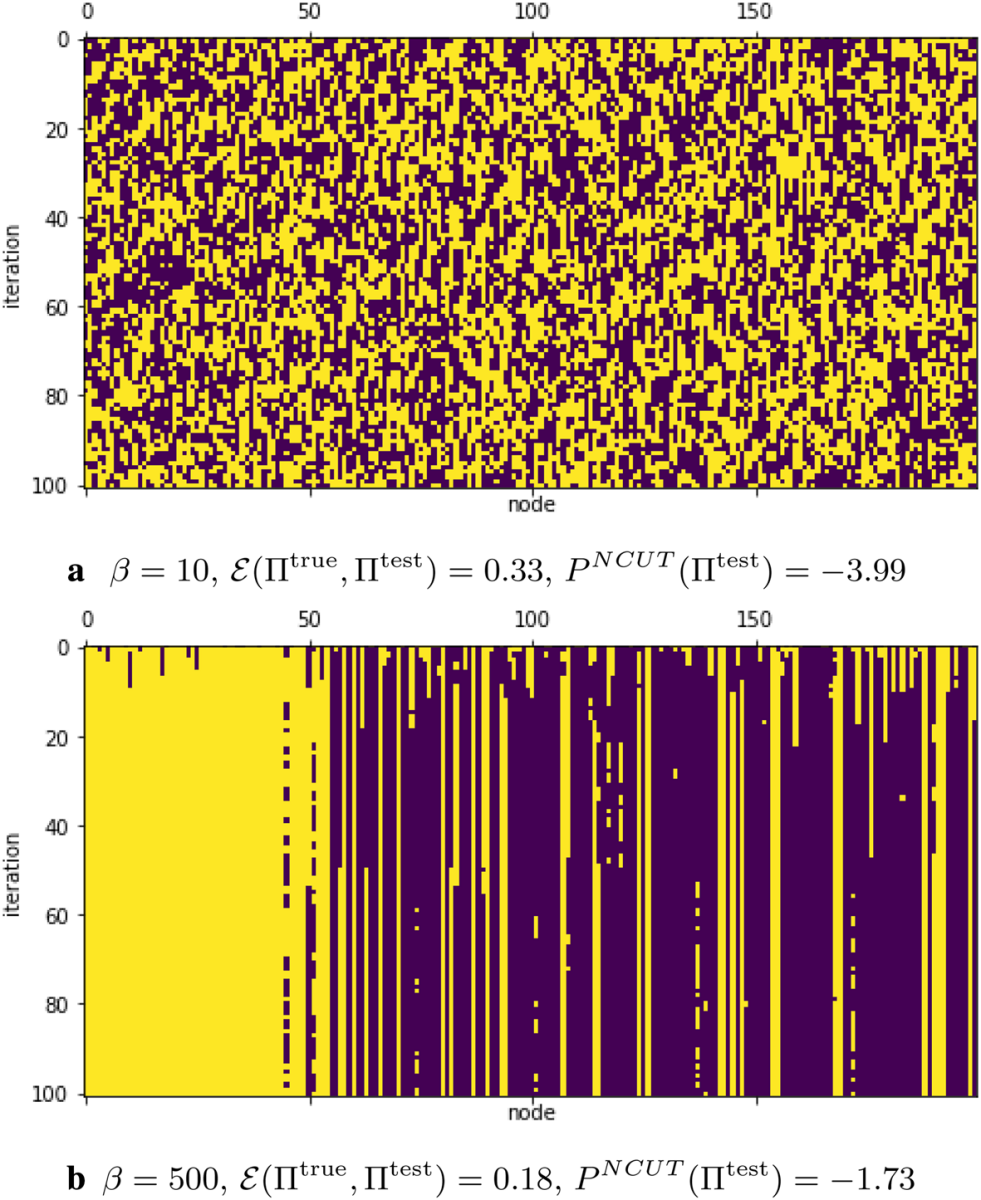
The iterations of NCUT-based Glauber dynamics for different $\beta$; $P^\text{NCUT}(\Pi ^{\text {true}}) = -\,1.40$. *x*-axis corresponds to the node index and *y*-axis corresponds to the iteration number. Different colors correspond to different clusters

In Fig. 5, we show an example of the Glauber dynamics using NCUT potential (12). For small $\beta = 10$ we observe unstable behavior, while for large $\beta = 500$ the process evolves around a local maximum that provides relatively bad clustering (49 out of 50 nodes of the first community and only 116 out of 150 of the second community are clustered correctly).

**Fig. 6:**
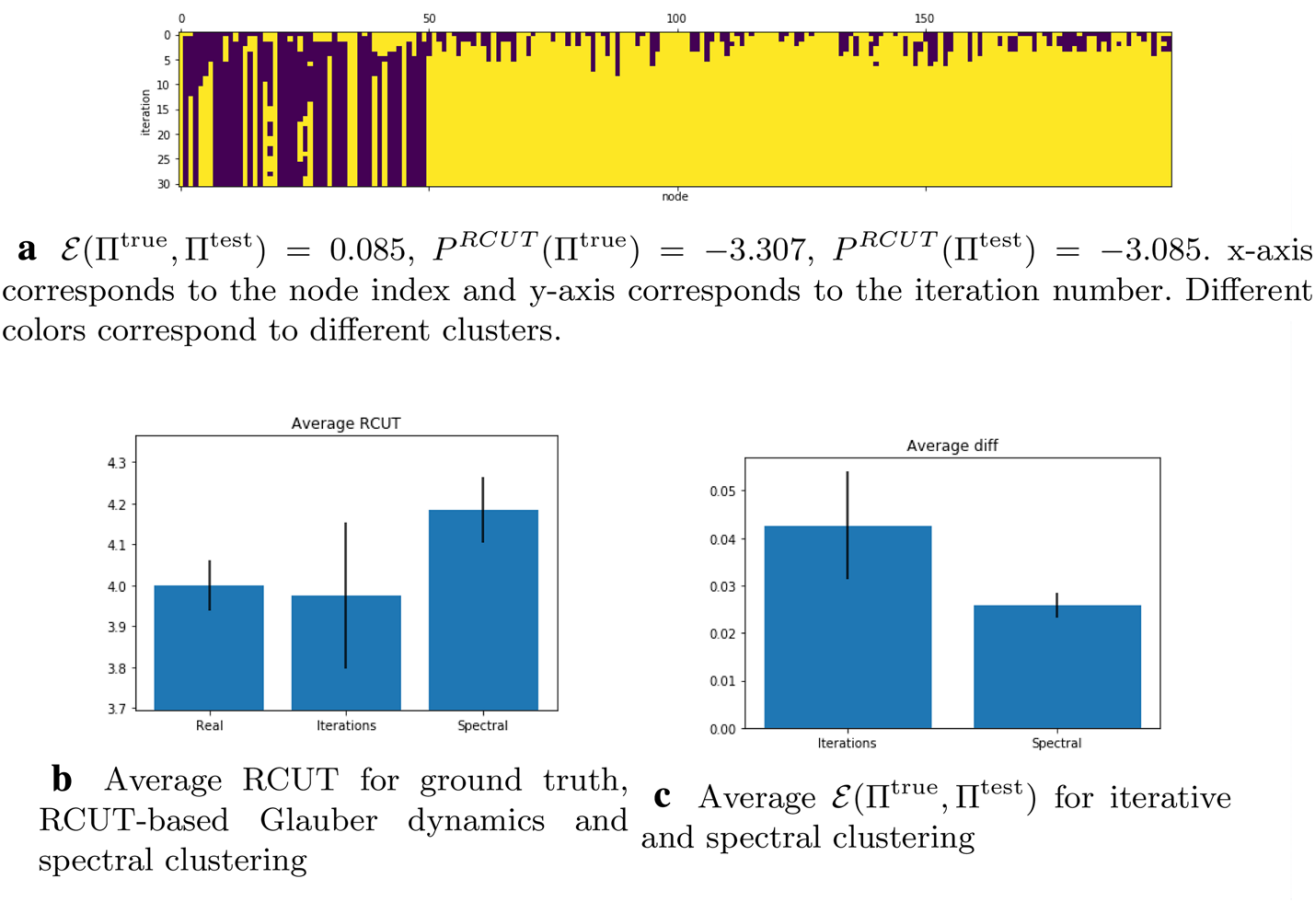
The iterations of RCUT-based Glauber dynamics for $\beta = 200$ and comparison to the ground truth and spectral clustering

Now let us take a closer look at RCUT (13). We discovered that in our example the ground truth partition does not maximize the potential. The process converges fast to a clustering that differs from the ground truth and has larger $P^{\text {RCUT}}$ than the ground truth partition. We tested the algorithm on a set of 100 graph instances generated according to the SBM and we show the results in Fig. 6 where we also compare it to the spectral clustering from [47]. One can see that while the Glauber dynamics generally ends up with $P^\text{RCUT}(\Pi ^{\text {test}}) > P^\text{RCUT}(\Pi ^{\text {true}})$. The spectral clustering procedure provides a solution that has smaller $P^{\text {RCUT}}$ but is closer to the ground truth.

**Fig. 7:**
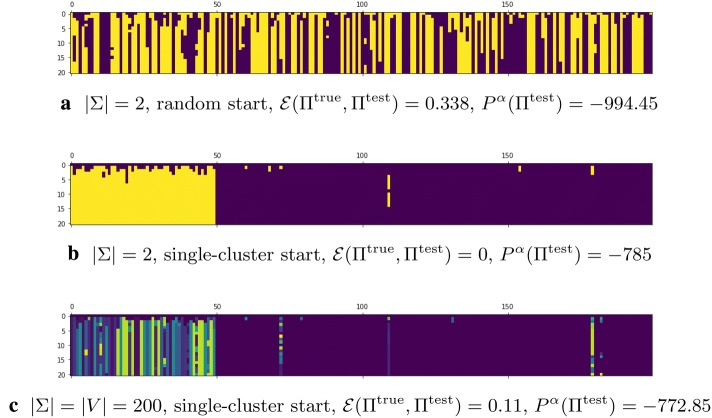
The iterations of hedonic-based process for $\alpha = 0.05$ and $\beta = 10$ for a graph with $P^\alpha (\Pi ^{\text {true}}) = -\,785$. *x*-axis corresponds to the node index and *y*-axis corresponds to the iteration number. Different colors correspond to different clusters

Next let us evaluate the performance of the clustering based on the potential $P^\alpha$, see (7). Empty clusters do not cause any singularities in $P^\alpha$ unlike in $P^\text{NCUT}$ and $P^\text{RCUT}$. Hence, the final partition can have less clusters than $|\Sigma |$. Let us at first restrict the number of clusters by setting $\Sigma = \{0, 1\}$. In this context, we have two natural choices for initial coloring of a graph: either, as before, we can choose colors uniformly at random, or we can assign same color to all nodes. We tested both settings on a set of 100 SBM graph instances. The best results are obtained with $\alpha = 0.05$ and $\beta = 10$. If we assign clusters at random at initialization, the process may not converge to a good coloring. Assigning the same initial color to all the nodes leads to better results. Fig. 7a and b shows evolution on the same graph with different initial colorings. The average ${\mathcal {E}}$ after 20 iterations for random-cluster initialization is 0.033, for single-cluster initialization it is 0.006; while the standard spectral clustering, i.e., the continuous relaxation of the NCUT [33] provides a result with average ${\mathcal {E}} (\Pi ^{\text {true}}, \Pi ^{\text {test}}) = 0.025$. We can conclude that the $P^\alpha$-based clustering significantly outperforms the spectral clustering in terms of accuracy. However, it depends on the parameter $\alpha$ that determines the penalty for large clusters. If $\alpha$ is too small, the uniform coloring becomes the ground state, as already indicated in Proposition 2. If $\alpha$ is too large, the obtained clusters will be relatively of the same size but may not represent the real community structure.

We can also try to detect the real number of clusters, if we choose large $\Sigma$. Here we can test the case when initially all nodes receive different colors $|\Sigma | = |V|$. We discovered that the final clustering consists of 9 or 10 clusters on average, most of which contain very few nodes. See Fig. 7c for an example of such clustering process.

To prevent the problem described in the previous paragraph, we modify $P^\alpha$ to $P^{\alpha ,\gamma }$, see Eq. (11), by introducing a penalty term proportional to the number of non-empty clusters. The potential $P^{\alpha ,\gamma }$ depends on parameters $\alpha$ and $\gamma$ that determine penalties for disparate clusters and for the total number of them, respectively. We tested the respective Glauber dynamics on the same set of random instances of SBM with parameters $\alpha = 0.05$, $\gamma = 5$, $\beta = 10$ , and $|\Sigma | = |V| = 200$. We run the process for 20 iterations and averaged the coloring over the last 10 of them. We obtained the following results: 2 clusters were determined in every graph instance and the average ${\mathcal {E}}(\Pi ^{\text {true}}, \Pi ^{\text {test}})$ is 0.0057. The average ${\mathcal {E}} (\Pi ^{\text {true}}, \Pi ^{\text {test}})$ for the spectral clustering is 0.0252.

In order to validate further the method based on $P^{\alpha ,\gamma }$-potential, we tried it on different sets of graphs of different clustering structures with the same algorithm parameters $\alpha$, $\gamma$ , and $\beta$. On a set of 100 homogeneous Erdős–Rényi random graphs of 200 nodes with edge density 0.1, our algorithm ended up with a uniform coloring on 99 of them and on one graph it finished with 2 clusters where the smaller one contains only two nodes. Given a set of 100 graph instances of SBM with clusters of 50, 150, and 200 nodes, the algorithm correctly determined the number of clusters in each graph and provided on average ${\mathcal {E}} (\Pi ^{\text {true}}, \Pi ^{\text {test}}) = 0.006$, while spectral clustering provided on average ${\mathcal {E}} (\Pi ^{\text {true}}, \Pi ^{\text {test}}) = 0.026$. On a set of 100 graph instances containing 4 clusters of 50, 100, 150, and 200 nodes, the algorithm after 20 iterations determined 4 clusters in 90 graphs and 3 clusters in 10 graphs. The average ${\mathcal {E}} (\Pi ^{\text {true}}, \Pi ^{\text {test}})$ is 0.0335. However, if we increase the number of iterations to 50, we determine correctly 4 clusters for 95 graphs and 3 clusters for the others. The average ${\cal {E}} (\Pi ^{\text {true}}, \Pi ^{\text {test}})$ becomes 0.0185. The average ${\mathcal {E}} (\Pi ^{\text {true}}, \Pi ^{\text {test}})$ of the spectral clustering on the same set of graphs is 0.0375.

The above results can be further improved by using the generalized covariance matrix. The application of the generalized covariance matrix will be illustrated in some of the following network examples.

#### Real-world network with ground truth: Karate club

**Fig. 8:**
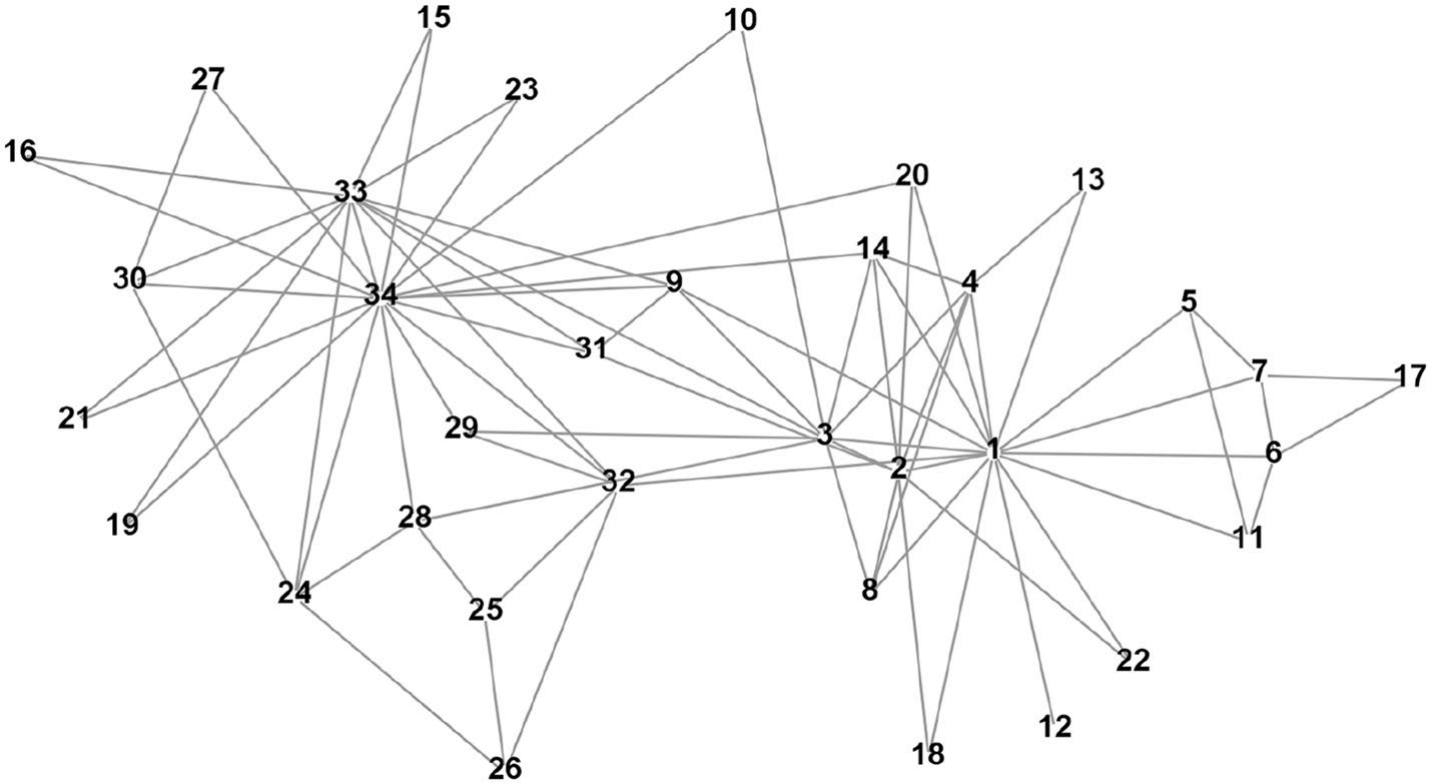
Zachary karate club network

Consider the popular example of the social network from Zachary karate club (see Fig. 8). In his study [49], Zachary observed 34 members of a karate club over a period of 2 years. Due to a disagreement developed between the administrator of the club and the club’s instructor there appeared two new clubs associated with the instructor (node 1) and administrator (node 34) of sizes 16 and 18, respectively.

**Fig. 9:**
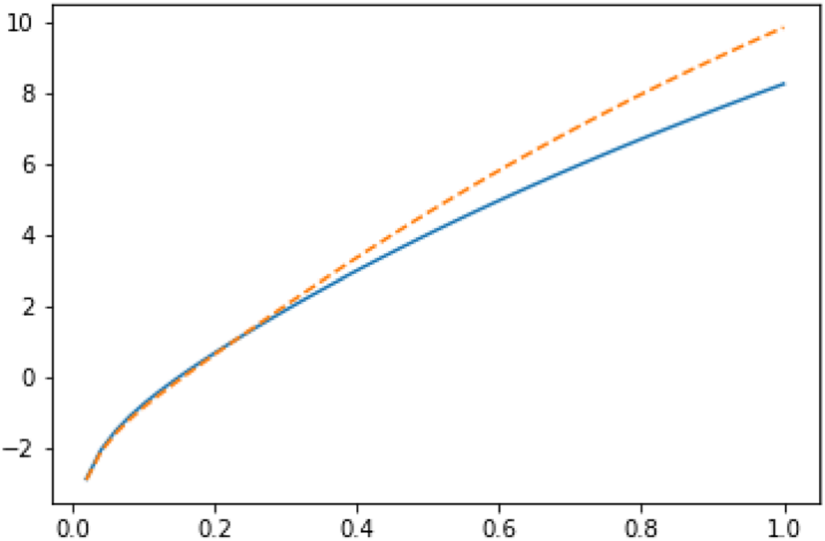
Imputations for node 3 in Zachary karate club network: $Y_3(g|R_3)$ (solid line) vs $Y_3(g|L_3 \cup \{3\})$ (dashed line) in semilog scale

**Fig. 10:**
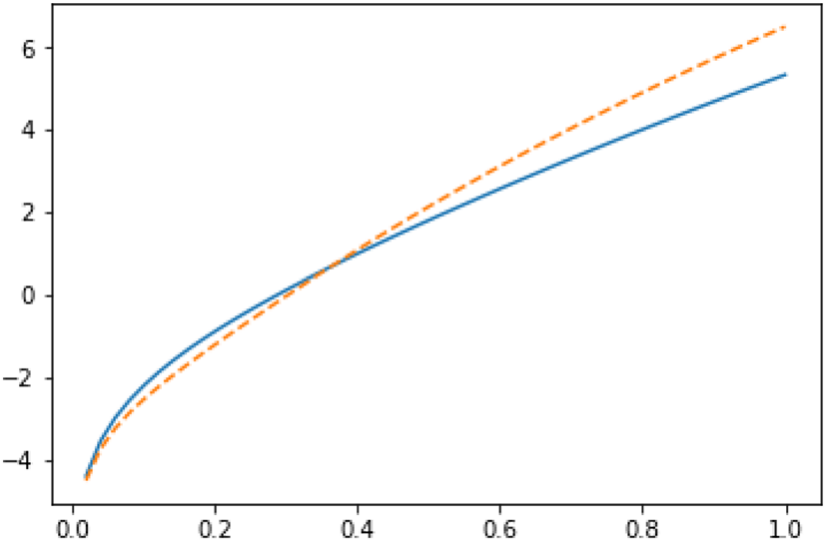
Imputations for node 10 in Zachary karate club network: $Y_{10}(g|L_3)$ (solid line) vs $Y_{10}(g|R_3 \cup \{10\})$ (dashed line) in semilog scale

**Fig. 11:**
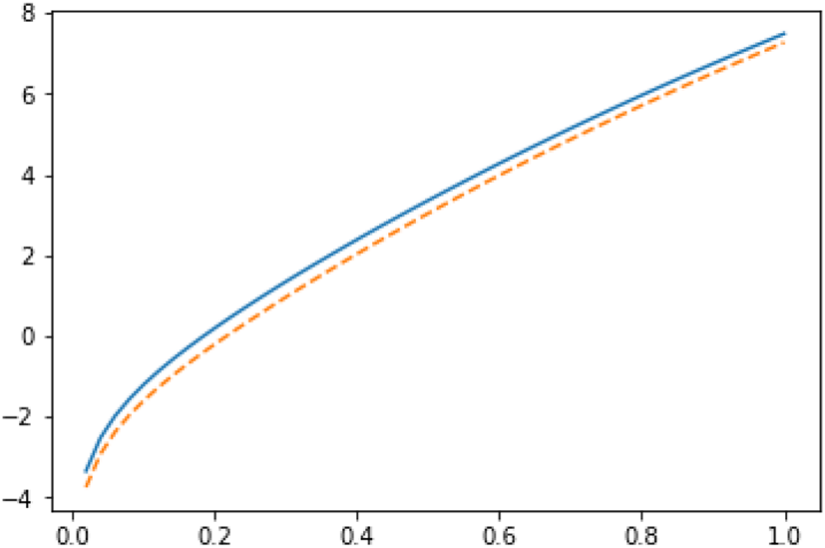
Imputations for node 9 in Zachary karate club network: $Y_{9}(g|L_3)$ (solid line) vs $Y_{9}(g|R_3 \cup \{9\})$ (dashed line) in semilog scale

The authors of [15] divide the network into two groups of roughly equal size using modularity and hierarchical clustering tree. They show that this split corresponds almost perfectly with the actual division of the club members following the break-up. Only one node, node 3, is classified incorrectly by the method of [15].

Let us first apply the Myerson value approach to the karate club network. To perform the analytic study, let us start from the ground truth partition [49]

$$\begin{aligned} R_3=\{1,2,3,4,5,6,7,8,11,12,13,14,17,18,20,22\} \quad \text{ and } \quad L_3=N\setminus R_3. \end{aligned}$$

By using subindex 3, we emphasize the importance of player 3. By enumerating all simple paths and using formula (4), we find the Myerson value for player 3 in coalition $R_3$

$$\begin{aligned} Y_3(g|R_3)= & {} \frac{5}{2}r+ \frac{41}{3}r^2+ \frac{224}{4}r^3+ \frac{883}{5}r^4+ \frac{2412}{6}r^5+ \frac{4378}{7}r^6+ \frac{5572}{8}r^7+ \\&\frac{6288}{9}r^8+ \frac{6040}{10}r^9+ \frac{3988}{11}r^{10}+ \frac{1392}{12}r^{11}+ \frac{120}{13}r^{12}, \end{aligned}$$

and in the coalition $L_3 \cup \{3\}$

$$\begin{aligned} Y_3(g|L_3 \cup \{3\})= & {} \frac{5}{2}r+ \frac{30}{3}r^2+ \frac{190}{4}r^3+ \frac{913}{5}r^4+ \frac{3426}{6}r^5+ \frac{8662}{7}r^6+\\&\frac{17286}{8}r^7+ \frac{29197}{9}r^8+ \frac{40452}{10}r^9+ \frac{40896}{11}r^{10}+ \frac{27080}{12}r^{11}+ \\&\frac{10701}{13}r^{12} + \frac{2209}{14}r^{13} + \frac{150}{15}r^{14}. \end{aligned}$$

We have plotted both $Y_3(g|R_3)$ and $Y_3(g|L_3 \cup \{3\})$ as functions of *r* in Fig. 9. If *r* is smaller than 0.231, node 3 has no incentive to move from coalition $R_3$ to coalition $L_3$. Recall that the modularity-based method of [15] would displace player 3 into the wrong coalition $L_3$.

It is also interesting to investigate the imputations of the other two border nodes 9 and 10. If we plot the imputations for node 10: $Y_{10}(g|L_3)$ and $Y_{10}(g|R_3 \cup \{10\})$ (see Fig. 10), we observe that as for node 3, for smaller values of *r* (i.e., for $r < 0.363$), node 10 has no incentive to leave the coalition $L_3$; whereas for the values of *r* greater than 0.363, node 10 has incentive to change the coalition.

As it is clear from Fig. 11, node 9 has no incentive to leave the coalition $L_3$ with any value of *r*. Thus, we can conclude that the ground truth partition [49] is internally stable according to the Myerson value approach if $r < 0.231$. This has a nice intuitive interpretation. Humans cannot count easily long paths and consequently one needs to apply heavy discounting to mimic humans’ decisions.

Let us now apply the hedonic game approach with Glauber dynamics to the karate club network. We started from a random partition into two clusters and run the algorithm using the potential (7) with $\alpha = 0.046$, which corresponds to 1/3 of the edge density, and $\beta = 20$. The algorithm stabilizes after around 5 iterations and the mean error after 10 iterations in 100 runs was 20.0%, which roughly corresponds 7 misclassified nodes. However, the partitioning results differ significantly from run to run.

By applying the spectral clustering algorithm from [47] to Zachary karate club network, we obtain an average error of 25.8%.

To reduce the variance of the Glauber dynamics and hence the clustering error, we computed the empirical generalized covariance matrix $\hat{M}$ for the results of 10 independent runs of 10 iterations of the Glauber dynamics and then extracted the community structure using the PCA algorithm. Only node 9 was misclassified, which is a border node.

The application of the generalized covariance matrix in addition to the Gibbs sampling really helps to consistently obtain high-quality results.

We would like to note that the application of the generalized covariance matrix to the spectral clustering method from [47] does not improve significantly its results since spectral clustering gives less noisy, however, more biased results compared to the hedonic game approach.

#### Real-world network with ground truth: Dolphins

**Fig. 12:**
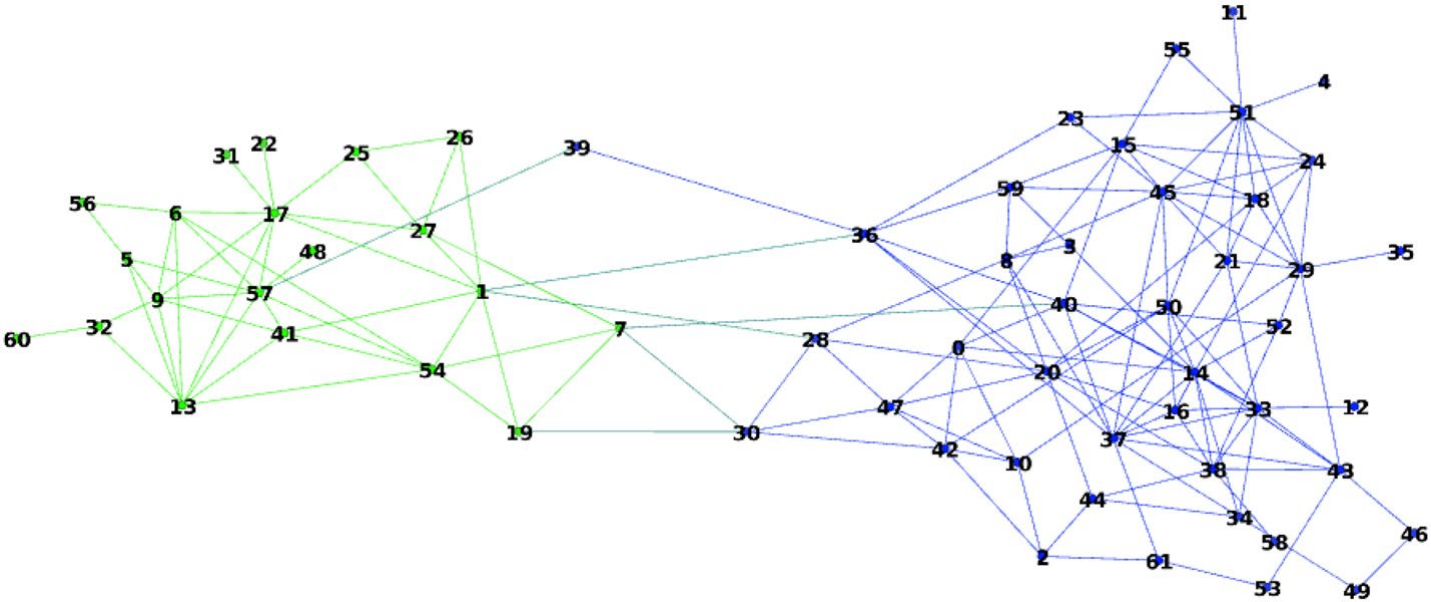
Dolphins social network

Consider now the Dolphins social network from [50]. This network presented in Fig. 12 was constructed from observations of a community of 62 bottle nose Dolphins over a period of 7 years from 1994 to 2001. The nodes in the network represent the Dolphins, and the ties between nodes represent the associations between dolphin pairs occurring more often than expected by chance.

The ground truth partition is presented by two coalitions:

$$\begin{aligned} L=\{1, 5, 6, 7, 9, 13, 17, 19, 22, 25, 26, 27, 31, 32, 41, 48, 54, 56, 57, 60 \} \end{aligned}$$

and

$$\begin{aligned} R\;=\; & {} \{0, 2, 3, 4, 8, 10, 11, 12, 14, 15, 16, 18, 20, 21, 23, 24, 28, 29, 30, 33, 34,\\&35, 36, 37, 38, 39, 40, 42, 43, 44, 45, 46, 47, 49, 50, 51, 52, 53, 55, 58, 59, 61\}. \end{aligned}$$

We studied the Dolphins network using the hedonic game approach with Glauber dynamics in a similar way as we did for Zachary karate club. Note that because of a very large number of simple paths in this network, the application of the Myerson value approach was not feasible. The following parameter values were used: $\alpha = 0.028$, which corresponds to 1/3 of the edge density, and $\beta = 20$. The algorithm stabilizes after around 10 iterations and the mean error after 20 iterations in 100 runs was 24.8%. As with Zachary karate club, the partitioning results differ significantly from run to run.

By applying the spectral clustering algorithm from [47], we obtain an average error of 7.5%.

We computed the empirical generalized covariance matrix $\hat{M}$ of the results of 10 independent runs of 20 iterations of the Glauber dynamics and then extracted the community structure using the PCA algorithm. Then, only one node, node 39, was misclassified. This is a border node connected to only two nodes of different clusters.

In contrast, computing the covariance matrix of 10 results of independent runs of spectral clustering algorithm from [47] led to 4 misclassified nodes: 22, 31, 48, 60.

#### Real-world network with many clusters and ground truth: Football

**Fig. 13:**
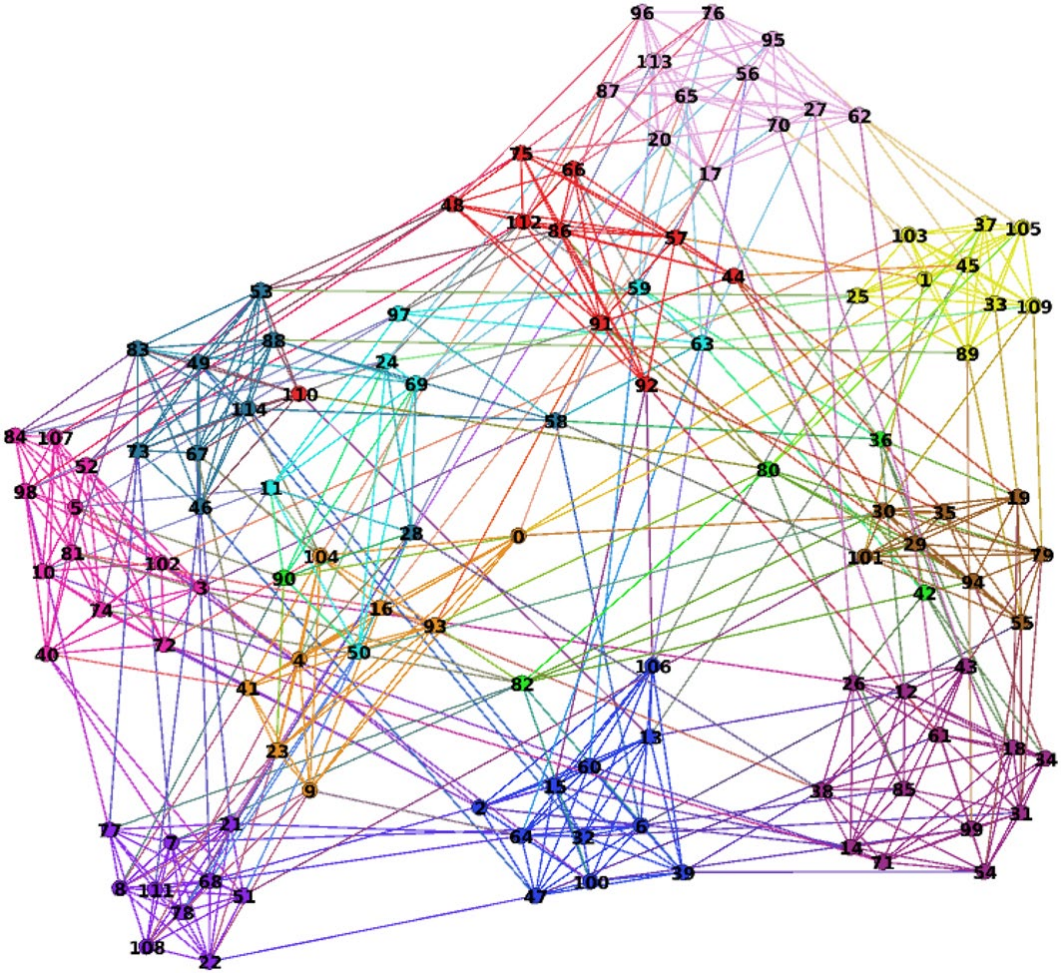
Football network

The American College Football network [15] represents the games between Division IA Colleges during the regular fall 2000 season (see Fig. 13).

The nodes in the network represent the teams and the links are the games between the teams. There are 115 teams and each team belongs to one of the 12 conferences. The communities in the network are the conferences. So, the ground truth partition is given by 12 coalitions:

$$\begin{aligned} C_1\;=\; & {} [112,48,92,44,75,66,91,86,57,110]; C_2\;=\;[50,24,69,11,97,59,63];\\ C_3\;=\; & {} [80,82,42,36,90]; C_4\;=\;[45,109,37,25,1,33,103,105,89];\\ C_5\;=\; & {} [9,4,104,0,93,16,41,23]; C_6\;=\;[5,74,3,98,10,40,84,81,52,102,107,72];\\ C_7\;= \;& {} [68,108,78,77,8,51,21,22,7,111]; C_8\;=\;[32,6,13,47,39,100,15,106,64,60,2];\\ C_9\;=\; & {} [61,99,43,14,12,31,38,18,54,34,71,26,85]; C_{10}\;=\;[29,19,94,35,55,30,101,79];\\ C_{11}\;=\; & {} [46,67,73,83,114,88,53,49,58,28]; C_{12}\;=\;[62,65,95,17,70,56,113,27,87,20,76,96]. \end{aligned}$$

As was the case of the Dolphins network, we could not apply the Myerson value approach to the football network because of the difficulty in enumerating all simple paths. In contrast, the hedonic game approach can easily be applied.

One of the main advantages of the hedonic game approach with $P^{\alpha , \gamma }$ potential (11) is the fact that it does not require the number of clusters as a parameter. Let us test on the football network, which consists of 12 clusters, how the hedonic game approach with $P^{\alpha , \gamma }$ potential can perform without a priori knowledge of the number of clusters.

We set the following parameters of the potential: $\alpha = 0.093$ (edge density), $\gamma = 10$. We run the Glauber dynamics with random initial partition into 20 clusters and the inverse noise $\beta = 10$ for 20 iterations. The clustering dynamics stabilizes after around 10 iterations. We performed 50 independent runs. The average number of detected clusters was 10.22 and the average percentage of misclassified nodes is 13.5%.

As with Zachary and Dolphins networks, we computed the empirical generalized covariance matrix of the clustering results. Since our goal was to determine the clusters without providing its number to the algorithm, we used a simple threshold-based clustering algorithm instead of the PCA algorithm: we build a weighted graph using the generalized covariance matrix as its adjacency matrix and removed the edges with weights below 0.5; the connected components of the resulting graph indicate clusters in the original network.

The resulting graph contained 13 connected components, i.e., we identified 13 clusters in the initial network that is quite close to the ground truth value 12. The percentage of the misclassified nodes is 6.9%, which signifies that the generalized covariance matrix improved significantly the quality of the clustering results.

#### Large real-world network without ground truth: Co-authorships in Math-Net.ru

To test scalability and efficiency of the hedonic game approach, we have chosen to cluster a fairly large social network. We have crawled the site Math-Net.ru, Russian Mathematical Portal, for the co-authorship graph [51]. We further extracted the giant connected component of this co-authorship network, which includes 41,840 authors. We have applied the hedonic game with the potential (7) and run the modified Glauber dynamics using round robin node schedule with random permutation. Twenty iterations of the modified Glauber dynamics run for about 2 min on Intel Core Duo 1.6 GHz processor and 5GB RAM. We have initialized process with a single cluster and restricted the number of clusters to ten. We have again observed stabilization of the modified Glauber dynamics at 15–20 iterations. Recall that one iteration of the modified Glauber dynamics requires *O*(|*E*|) operations, which is quite a reasonable cost in the case of sparse networks and which is the case of most real-world networks. We have chosen significantly high value of $\beta$, which corresponds to nearly greedy algorithm. First, we have run the modified Glauber dynamics with $\alpha$ corresponding to the average edge density. This has lead to unbalanced clusters, see Table 1. By expecting clusters, we have observed that nearly all academicians (aka leaders of scientific schools) have been clustered to one largest cluster. However, when we have increased $\alpha$ tenfold, the clustering became much more balanced and the academicians have been distributed more evenly among the clusters.

**Table 1:** Cluster sizes in Math-Net.ru

$\alpha$	$|S_1|$	$|S_2|$	$|S_3|$	$|S_4|$	$|S_5|$	$|S_6|$	$|S_7|$	$|S_8|$	$|S_9|$	$|S_{10}|$
0.000112	16,457	2820	2820	2820	2821	2821	2820	2821	2820	2820
0.001124	4184	4184	4184	4185	4183	4184	4184	4184	4184	4184

#### Large synthetic SBM network with ground truth

To continue testing scalability and efficiency of the hedonic game approach and in particular to confirm a rapid convergence of the Glauber dynamics to a good solution, we consider a large stochastic block model graph with known communities. Specifically, we have generated an SBM with two clusters of sizes 50,000 and 150,000 nodes. We have generated the intra-cluster links with probability 0.0002 and the inter-cluster links with probability 0.00005. We run the Glauber dynamics associated with the potential (7), setting $\alpha = 0.0001$ and $\beta = 10$. In a typical run, after 7 iterations, only 97 nodes from the smaller cluster were misclassified to the larger cluster and only 65 nodes from the larger cluster were misclassified to the smaller cluster. It is not surprising that by the “gravity” effect the larger cluster attracted more nodes. We find that 7 iterations of the Glauber dynamics are not at all a large cost for partitioning 200,000 node network.

## Conclusion and future research

We have presented two cooperative game theory-based approaches for network partitioning. The first approach is based on the Myerson value for graph constrained cooperative game, whereas the second approach is based on hedonic games which explain coalition formation. We find the second approach especially interesting as it gives a very natural way to tune the clustering resolution and generalizes the modularity, ratio cut, and normalized cut-based approaches. Within the hedonic games framework, we have proposed two new methods which particularly well regularize clustering resolution and help to adjust the level of granularity. We have shown that normalized cut and ratio cut methods can be modified to avoid the requirement of the number of clusters. All approaches that can be represented as hedonic games with potentials can be very efficiently implemented using Gibbs sampling with Glauber dynamics and generalized covariance matrix. The application of the generalized covariance matrix significantly improves the quality and stability of the clustering results. Our research plans are to test and to compare our methods on more social networks and to study analytically the convergence rate of Gibbs sampling.
